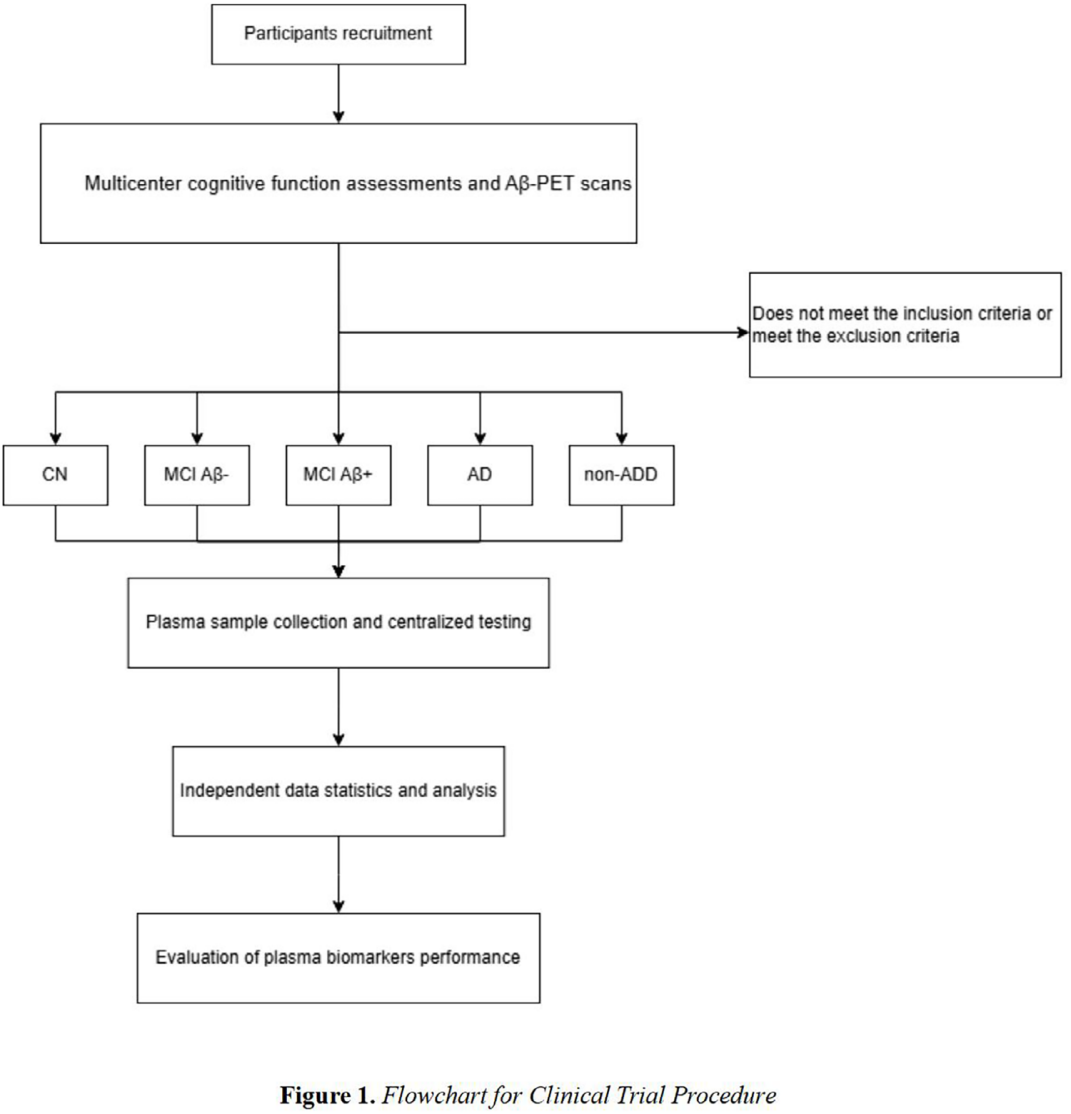# Clinical Evaluation of Blood‐Based Assays for Rapid Detection of Amyloid‐β Pathology in Alzheimer's Disease (CLEAR‐AD)

**DOI:** 10.1002/alz70856_100197

**Published:** 2025-12-24

**Authors:** Xinyi Lyu, Feng Gao, Xiaodong Pan, Guoping Peng, Shuting Z, Qianhua Zhao, Fang Xie, Huayan Liu, Haining Zhang, Wei Chen, Mengya Xing, Yuesong Pan, Qidong Chen, Yiming Wang, Zengshi Lin, Xiaochun Chen, Yong Shen, Jiong Shi

**Affiliations:** ^1^ Department of Neurology, The First Affiliated Hospital of USTC, Division of Life Sciences and Medicine, University of Science and Technology of China, Hefei, Anhui, China; ^2^ Department of Neurology, Institute on Aging and Brain Disorders, The First Affiliated Hospital of USTC, Division of Life Sciences and Medicine, University of Science and Technology of China, Hefei, Anhui, China; ^3^ Department of Neurology, Fujian Medical University Affiliate Union Hospital, Fuzhou, Fujian, China; ^4^ First Affiliated Hospital, Zhejiang University, Hangzhou, China; ^5^ Department of Neurology and State Key Laboratory of Biotherapy, West China Hospital, Chengdou, Sichuan, China; ^6^ Institute of Neurology, Huashan Hospital, Fudan University, Shanghai, China; ^7^ Huashan Hospital, Fudan University, Shanghai, Shanghai, China; ^8^ Department of Neurology, the First Hospital of China Medical University, Shenyang, liaoning, China; ^9^ Department of Neurology and Neuroscience Center, The First Hospital of Jilin University, Jilin University, Changchun, jilin, China; ^10^ Department of Physiology and Department of Psychiatry, Sir Run Run Shaw Hospital, Zhejiang University School of Medicine, hangzhou, zhejiang, China; ^11^ Department of Neurology, Tianjin Neurological Institute, Tianjin Medical University General Hospita, tianjin, tianjin, China; ^12^ Beijing Tiantan Hospital, Capital Medical University, beijing, beijing, China; ^13^ Beijing Tiantan Hospital, Capital Medical University, Beijing, China; ^14^ Eisai China Inc., Shanghai, Shanghai, China; ^15^ Eisai China Inc., Shanghai, China; ^16^ Neurodegenerative Disorder Research Center, Division of Life Sciences and Medicine, University of Science and Technology of China, Hefei, Anhui, China

## Abstract

**Background:**

Blood‐based biomarkers show promise in predicting Alzheimer's disease (AD) pathology and progression; however, inconsistencies in detection standards hinder clinical application. A head‐to‐head comparison of commercially available biomarkers is crucial for optimizing the clinical pathway for AD screening and diagnosis.

**Method:**

The CLEAR‐AD study is an ongoing population‐based cross‐sectional study, currently recruiting 400 participants in ten centers in China. The study includes cognitively normal controls, individuals with mild cognitive impairment (MCI) — categorized as amyloid‐positive and amyloid‐negative — as well as patients with dementia, also divided into amyloid‐positive and amyloid‐negative groups. All participants undergo amyloid PET scans using tracers such as AV1, AV45, and PIB. Blood samples are collected within three months prior to the PET scan or from existing samples collected after January 1, 2024, that meet quality standards. After collection, these samples are analyzed at a central laboratory under blinded conditions using multiple detection methods to measure plasma levels of Aβ40, Aβ42, t‐tau, and *p*‐tau181/217. The detection technologies included single‐molecule immunoassay, digital immunoassay chips, magnetic particle chemiluminescence, and flow cytometry fluorescence. The objective is to assess the sensitivity and specificity of different plasma biomarker levels in predicting amyloid pathology confirmed by Aβ‐PET. A flowchart illustrating the study process is shown in Figure 1.

**Result:**

The study uses Aβ‐PET as the reference standard to evaluate the sensitivity and specificity of various AD plasma biomarkers across different detection methods in diagnosing amyloid pathology. The analysis included generating receiver operating characteristic (ROC) curves, determining optimal cut‐off values, and developing a predictive model that integrates multiple biomarker parameters and clinical data. Results is considered statistically significant with a *p*‐value of less than 0.05.

**Conclusion:**

CLEAR‐AD study provided crucial data on the effectiveness of commercially available blood biomarkers for predicting amyloid pathology and inform the optimization of early AD screening in the Chinese population. The findings will guide future developments in diagnostic tools.